# Effective Fundus Image Decomposition for the Detection of Red Lesions and Hard Exudates to Aid in the Diagnosis of Diabetic Retinopathy

**DOI:** 10.3390/s20226549

**Published:** 2020-11-16

**Authors:** Roberto Romero-Oraá, María García, Javier Oraá-Pérez, María I. López-Gálvez, Roberto Hornero

**Affiliations:** 1 Biomedical Engineering Group, Universidad de Valladolid, 47011 Valladolid, Spain; maria.garcia@tel.uva.es (M.G.); javier.oraa@gib.tel.uva.es (J.O.-P.); maribel@ioba.med.uva.es (M.I.L.-G.); roberto.hornero@tel.uva.es (R.H.); 2 Centro de Investigación Biomédica en Red de Bioingeniería, Biomateriales y Nanomedicina (CIBER-BBN), 28029 Madrid, Spain; 3 Department of Ophthalmology, Hospital Clínico Universitario de Valladolid, 47003 Valladolid, Spain; 4 Instituto Universitario de Oftalmobiología Aplicada (IOBA), Universidad de Valladolid, 47011 Valladolid, Spain; 5 Instituto de Investigación en Matemáticas (IMUVA), Universidad de Valladolid, 47011 Valladolid, Spain

**Keywords:** diabetic retinopathy, fundus image, retinal decomposition into layers, red lesions, exudates

## Abstract

Diabetic retinopathy (DR) is characterized by the presence of red lesions (RLs), such as microaneurysms and hemorrhages, and bright lesions, such as exudates (EXs). Early DR diagnosis is paramount to prevent serious sight damage. Computer-assisted diagnostic systems are based on the detection of those lesions through the analysis of fundus images. In this paper, a novel method is proposed for the automatic detection of RLs and EXs. As the main contribution, the fundus image was decomposed into various layers, including the lesion candidates, the reflective features of the retina, and the choroidal vasculature visible in tigroid retinas. We used a proprietary database containing 564 images, randomly divided into a training set and a test set, and the public database DiaretDB1 to verify the robustness of the algorithm. Lesion detection results were computed per pixel and per image. Using the proprietary database, 88.34% per-image accuracy (ACC_i_), 91.07% per-pixel positive predictive value (PPV_p_), and 85.25% per-pixel sensitivity (SE_p_) were reached for the detection of RLs. Using the public database, 90.16% ACC_i_, 96.26% PPV__p_, and 84.79% SE_p_ were obtained. As for the detection of EXs, 95.41% ACC_i_, 96.01% PPV__p_, and 89.42% SE__p_ were reached with the proprietary database. Using the public database, 91.80% ACC_i_, 98.59% PPV_p_, and 91.65% SE_p_ were obtained. The proposed method could be useful to aid in the diagnosis of DR, reducing the workload of specialists and improving the attention to diabetic patients.

## 1. Introduction

Diabetic retinopathy (DR) is a microvascular complication of diabetes mellitus [1,2]. While it is asymptomatic in its initial stage, it leads progressively to vision loss [2]. With the rising incidence of diabetes, DR has become the main cause of blindness and visual impairment in the working age population [1]. However, it is proven that serious sight damage can be prevented through early, accurate diagnosis and proper eye care [1,3]. Therefore, it is important to carry out regular DR examinations based on the analysis of color fundus images to detect the characteristic signs of the DR: red lesions (RLs), such as microaneurysms (MAs) and hemorrhages (HEs), and bright lesions, such as exudates (EXs) [2,4]. MAs, as the earliest visible sign of DR, are caused by leakages of tiny blood vessels. They appear as reddish, small, and circular dots. HEs are produced by retinal ischemia and rupture of damaged and fragile retinal vessels. They generally look like bigger red spots with irregular shapes. EXs are fluid deposits of lipoproteins and other proteins leaking through abnormal retinal blood vessels. They appear as yellowish, bright patches of varied shapes and sizes with sharp edges [2,4]. Although manual reading of retinal images has proven effective in patient care, it requires a lot of effort, time, and costs [5]. In this context, computer-assisted diagnostic systems (CADSs) are designed to relieve the workload of specialists, reduce the health costs, and hasten the diagnosis [1,4].

Several studies have been developed in the past twenty years to detect DR-related lesions in fundus images [6]. Some approaches focused on detecting Mas alone and can be divided into four groups [4]: mathematical morphology-based [7], region growing-based [8], wavelet-based [9], and hybrid approaches [10,11]. Other methods focused on exclusively detecting HEs and can be divided into two categories [4]: mathematical morphology [12,13] and pixel classification [14]. However, both MAs and HEs are typically detected together, because they frequently look similar and their distinction is not necessary to determine the presence of DR [1]. This way, we can find several works where all RLs are detected together. Among the recent studies, Seoud et al. [15] proposed a technique based on dynamic shape features that represent the evolution of the shape during image flooding. Other authors divided the image into superpixels to detect RLs [16,17]. Srivastava et al. [18] proposed to apply filters on patches of different sizes and to combine the results using multiple kernel learning. Deep learning has also been employed in some studies, achieving successful results in the detection of RLs [19,20,21].

Numerous methods have also been proposed to detect EXs. Generally, they can be divided into three groups: clustering-based [22,23,24]; mathematical morphology, thresholding, and region growing-based [25,26,27]; and pixel classification-based [28,29]. Among the most recent studies, an unsupervised approach based on the ant colony optimization algorithm has been proposed [30]. Theera-Umpon et al. [31] proposed an EX detection method exploring other techniques of supervised learning, such as support vector machines and neural networks. Superpixel approaches can also be found in the literature [32]. Other authors have employed deep learning together with some additional operations [33,34,35]. In this context, Guo et al. [36] introduced a novel top-*k* loss for EXs segmentation, which considers class-unbalance and loss-unbalance by focusing more on the hard-to-classify pixels.

None of the previous studies have individually considered other structures of the retina beyond the optic disc (OD), the fovea, and the vasculature. We hypothesize that the reflective features of the retina and the choroidal vasculature visible in tigroid retinas can also be useful for the detection of retinal lesions. In this study, we propose a novel method to detect RLs and EXs where the image is decomposed into several layers providing, separately, information on different structures of the retina. Among these layers, the lesion candidates, the reflective features, and the choroidal vessels were included, which is the main contribution of this work. This decomposition was based on human perception. Hence, the layers were directly separated using color and spatial information.

## 2. Retinal Image Databases

In this study, both a proprietary dataset and a public database were employed. The proprietary dataset was divided into a training set, used for the development of the method, and an independent test set, used to evaluate the performance. We also used the public database DiaretDB1 [37] to verify the robustness and generalization ability of the proposed method.

The proprietary dataset consisted of 564 retinal images provided by the Instituto de Oftalmobiología Aplicada (IOBA) of the University of Valladolid (Valladolid, Spain) and the Hospital Clínico Universitario de Valladolid (Valladolid, Spain). All subjects gave their informed consent to participate in the study. Our research was conducted in accordance with the Declaration of Helsinki, and the protocol was approved by the Ethics Committee at the Hospital Clínico Universitario de Valladolid. The images were captured using a Topcon TRC-NW400 automatic retinal camera (Topcon Medical Systems, Inc., Oakland, NJ, USA) at a 45-degree field of view (FOV). All images had a resolution of 1956 × 1934 pixels and were captured using the two-field protocol adopted by National Service Framework for Diabetes in the United Kingdom for DR screening [38]. This way, two images were captured per eye: one fovea-centered and one OD-centered. It should be noted that, for some patients, fewer than four images were available. All the RLs and EXs in this database were manually annotated in detail by an ophthalmologist. While 270 out of 564 fundus images showed DR signs, the remaining 294 lacked any type of lesion. Among the 270 pathological images, 183 showed EXs, 239 showed RLs, and 152 images included both EXs and RLs. The database was randomly divided into two balanced sets. The training set (281 images) allowed us to develop the method and optimize its parameters. The test set (283 images) was used to evaluate the performance of the method. It should be noted that all the images of the same patient were included in the same set.

The DiaretDB1 database is composed of 89 images captured in the Kuopio University Hospital and divided into a training set (28 images) and a test set (61 images) [37]. They were captured at a 50-degree FOV and had a resolution of 1500 × 1552 pixels. All the images were fovea-centered, and the ground truth of the lesions was roughly annotated by four experts using circles, ellipses, and polygons. The DiaretDB1 database was used to verify the robustness of the proposed method and compare our results with those obtained in previous studies. In order to perform this comparison, only the 61 images in the test set of DiaretDB1 were used.

## 3. Methods

### 3.1. Overview

**Figure 1:**
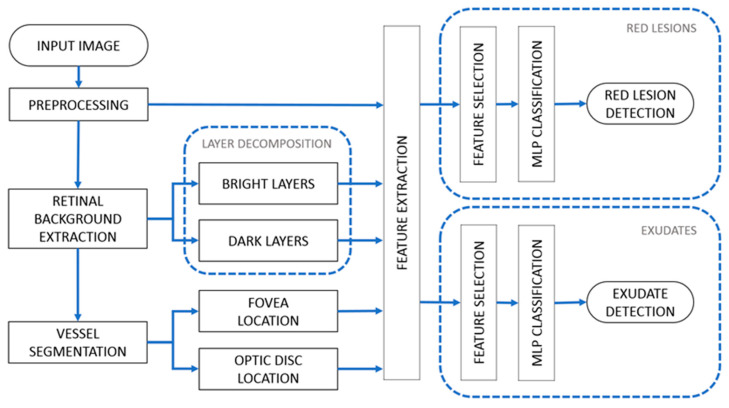
Diagram of the proposed method. (1) Preprocessing. (2) Retinal background extraction. (3) Vessel segmentation, optic disc location, and fovea location. (4) Layer decomposition. (5) Feature extraction and selection. (6) Multilayer perceptron (MLP) classification.

The proposed method comprises several steps. First, we applied a preprocessing stage to normalize the image appearance and enhance the retinal structures. Second, we obtained an estimation of the retinal background, the segmentation of the vasculature, and the location of the OD and the fovea. Third, the image was decomposed into several layers. Then, multiple lesion candidates were segmented. Next, several features were extracted using the obtained layers, and feature selection was performed using the fast correlation-based filter (FCBF) technique [39]. Finally, a multilayer perceptron (MLP) was used to distinguish the true lesions from the rest of the candidates [40]. All the steps are described in the following subsections. The diagram in Figure 1 shows the global structure of the proposed method.

### 3.2. Preprocessing

**Figure 2:**
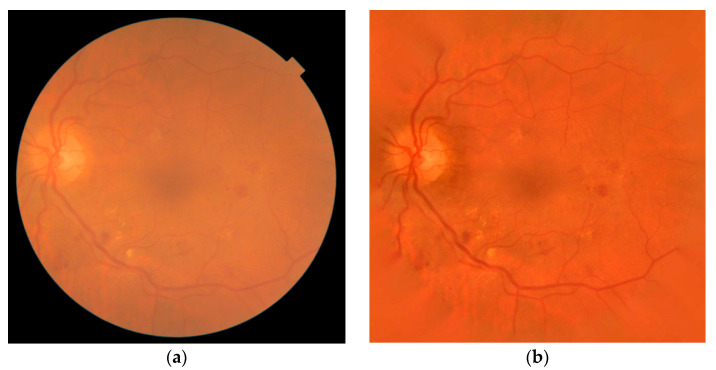
Preprocessing stage. (**a**) Original image. (**b**) Preprocessed image, $I_{prep}$.

The appearance of the fundus images is strongly affected by the image quality and the intrinsic features of the patient [10,15]. A preprocessing stage is required to normalize the input images in order to make subsequent processing easier. In this study, we applied our method in [17], consisting of five sequential operations: bright border artifact removal, background extension, illumination and color equalization, denoising, and contrast enhancement. The retinal landmarks were notably highlighted and the intra-image and inter-image normalization was achieved. The result of this stage, $I_{prep}$, can be seen in Figure 2. From this stage, we also obtained the diameter of the FOV, *D*, which allowed us to obtain relative sizes for subsequent operations.

### 3.3. Background Extraction

**Figure 3:**
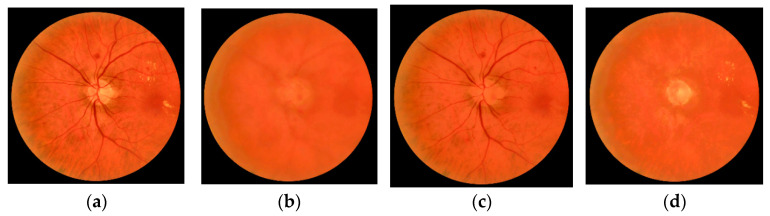
Background extraction stage. (**a**) Preprocessed image. (**b**) Estimated background, $I_{bg}$. (**c**) Estimated background preserving dark structures, $I_{bg-dark}$. (**d**) Estimated background preserving bright structures, $I_{bg-bri}$.

In order to detect the visible lesions in fundus images, it is important to separate the background and the foreground. The foreground covers the main information and is composed of dark pixels and bright pixels relative to the background [41]. In this stage, we estimated the background of the fundus image using our method in [42]. Additionally, we eliminated the dark pixels in $I_{prep}$ to obtain the image $I_{bg-bri}$ and eliminated the bright pixels to obtain the image $I_{bg-dark}$. The images obtained in this stage can be seen in Figure 3. For this figure, we selected a more appropriate example than the image in Figure 2.

### 3.4. Detection of the Main Anatomical Structures

The fovea, the OD, and the vascular network are the most important landmarks in the fundus image [43]. Since the appearance of the fovea and the blood vessels is dark, they can be confused with some RLs. In the same way, parts inside the OD can potentially be classified as EXs [43]. Therefore, the prior detection of these structures is useful for the automatic detection of retinal lesions. The aim of this stage was to segment the vasculature and locate the OD and fovea centers. For this task, we applied our methods in [42], which have proved robustness using four different databases. These methods properly work with different types of FOV and do not require a standardized imaging protocol. The vasculature segmentation, $M_{vess}$, was based on vessel centerlines detection, region growing, and morphology. The OD and fovea locations were based on various saliency maps. Morphological operations, spatial filters, and template matching were applied [42]. The OD was modeled as a circle with radius $R_{OD}=\frac{D}{12}$ pixels [44]. 

### 3.5. Red Lesion Candidate Segmentation

**Figure 4:**
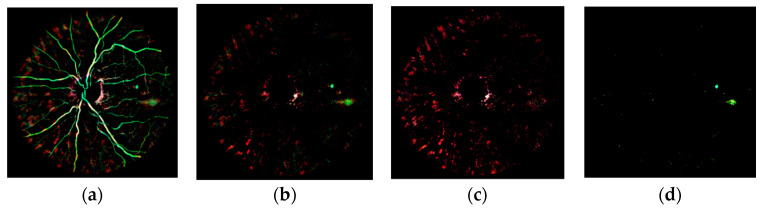
Red lesion candidate segmentation. (**a**) Image $I_{dark}$. (**b**) Image $I_{dark-2}$. (**c**) Image $L_{chor-dark}$. (**d**) Image $L_{rl-cand}$. These images are shown with enhanced contrast for an easier readability.

In order to detect RLs, the fundus image was decomposed into several layers. Each layer represented a different structure of the retina and provided useful information for the detection of retinal lesions. Since RLs appear as dark regions, we calculated the complement of the subtraction $I_{bg-dark}-I_{bg}$ in order to select the dark pixels, obtaining $I_{dark}$. In this image, the color difference between dark pixels and the background was highlighted, while leaving the rest of the pixels black (see Figure 4a).

After preprocessing, inter- and intra-image variability was reduced. Hence, the color of the visible structures in $I_{dark}$ was always consistent. This way, they could be directly separated using color and spatial information. The blood vessels, the choroidal atrophy, and the rest of dark pixels, which are RL candidates, are the structures of interest in $I_{dark}$ that should be taken into account for RL detection.

As expected, the color of the vasculature in $I_{dark}$ is very similar to the color of RLs. Therefore, we eliminated the pixels belonging to blood vessel using the mask $M_{vess}$ obtained in Section 3.4. The result, $I_{dark-2}$, can be seen in Figure 4b. There is another type of structure that is easily distinguishable in $I_{dark}$: the underlying choroidal vasculature visible in tigroid retinas [45]. This is caused by the lack of pigments in the retinal pigment epithelium and is common in aged or myopic patients [45]. It has pink tones in $I_{dark}$ and can hinder the detection of RLs due to the high contrast it shows against the background. Separating the choroidal vasculature is useful to classify the RLs in the image, since images featuring very marked choroidal vessels tend to present false positives [17]. For this reason, the next step was to separate the layer corresponding to the choroidal vessels in $I_{dark-2}$. For this task, we used the color information in the Hue-Saturation-Value (HSV) color space. This color space was designed to approximate the eye perception and is useful to replicate human interpretation [46]. It decouples the brightness component from the color-carrying information. Using HSV, we analyzed the pixels belonging to the choroidal vessels using the training set. This way, we empirically selected the ranges of values that represented those pixels for each channel. In this work, these ranges were determined to be H=[0.75, 0.1], S=[0.2, 1.0], and V=[0.05, 1.0]. Then, we segmented the pixels in $I_{dark-2}$ that were between the selected ranges, obtaining the image $L_{chor-dark}$, which represented the layer of choroidal atrophy (see Figure 4c). In the same way, we selected the HSV color ranges associated with RLs. For this task, we analyzed all the pixels belonging to RLs in the images of the training set annotated by the ophthalmologist. The selected ranges in this work were H=[0.1, 0.45], S=[0.1, 1.0], and V=[0.2, 1.0]. Then, we segmented the pixels in $I_{dark-2}$ that were between the selected ranges, obtaining the layer $L_{rl-cand}$, associated to potential RLs (see Figure 4d). Finally, the mask of potential RL candidates was obtained binarizing the layer $L_{rl-cand}$ in order to obtain the candidate binary mask $M_{rl-cand}$. The obtained layers of interest in this phase, $L_{chor-dark}$ (Figure 4c) and $L_{rl-cand}$ (Figure 4d), were also useful for lesion candidate classification in later stages.

### 3.6. Exudate Candidate Segmentation

**Figure 5:**
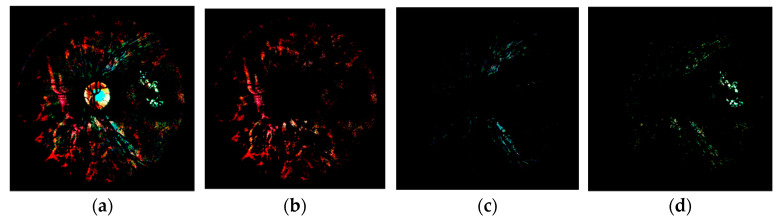
Exudate candidate segmentation. (**a**) Image $I_{bri}$. (**b**) Image $L_{chor-bri}$. (**c**) Image $L_{bm}$. (**d**) Image $L_{ex-cand}$. These images are shown with enhanced contrast for an easier readability.

EXs appear as bright regions in contrast with the background. Therefore, we subtracted the image $I_{bg}$ from the $I_{bg-bri}$ image in order to select the bright pixels. The obtained image, $I_{bri}$, enhanced the color difference of the bright pixels with respect to the retinal background, while leaving the rest of the pixels black (see Figure 5a).

Following the same idea as for the segmentation of RLs, $I_{bri}$ was divided into different layers of information. The proposed method relied in the idea that the color of the pixels in $I_{bri}$ for each type of structure is constant. This way, we separated the different structures of interest in $I_{bri}$ using color and spatial information. The HSV color space was also employed here [46]. The structures of interest in $I_{bri}$ are the choroidal vasculature, the reflective features, and the EX candidates.

Often, parts of the tigroid retina appear as bright pixels in contrast to the retinal background and are shown in red tones in $I_{bri}$ (see Figure 5a). We analyzed the pixels in $I_{bri}$ belonging to choroidal vessels in tigroid retinas using the training set. We empirically selected the ranges in HSV representing those pixels. In this study, those ranges were H=[0.75, 0.15], S=[0, 1.0], and V=[0, 1.0]. Then, the layer of choroidal vessels, $L_{chor-bri}$, was obtained segmenting the pixels in $I_{bri}$ that were between the selected ranges (see Figure 5b). Other structures visible in $I_{bri}$ were the reflective features caused by the nerve fiber layer [47]. They are very common when it comes to retinas in young patients and cannot be considered as abnormalities. Most of these marks are concentrated along the widest vessels [47] and tend to be green and blue in $I_{bri}$ (see Figure 5a). Due to their color, they can be confused with EXs. Therefore, separating the reflective features is useful to classify the true EXs in the image. In order to separate reflective features from lesions, we selected the pixels associated with reflective features using the HSV color space in the training set. The selected ranges were H=[0.25, 0.85], S=[0, 1.0], and V=[0, 1.0], obtaining the image $I_{bm1}$. On the other hand, we selected the pixels surrounding the main vessels in the vascular network. For this task, we first performed a morphological opening over the image $M_{vess}$ to roughly remove the thin vessels. A disk of radius $\frac{R_{OD}}{10}$ pixels was applied. Second, a morphological dilation was performed using a disk of radius $\frac{D}{60}$ pixels, obtaining the image $I_{bm2}$. Finally, the reflective features layer, $L_{bm}$, was obtained by multiplying $I_{bm1}$ and $I_{bm2}$ to select the bright marks surrounding the vasculature (see Figure 5c). Using the same idea, the layer of potential EXs was also extracted from $I_{bri}$. We used the HSV color space and, for each component, selected the ranges of values among which the EXs were. For this task, we analyzed all the pixels belonging to EXs in the images of the training set annotated by the ophthalmologist. In this work, the selected ranges were H=[0.15, 0.45], S=[0.1, 1.0], and V=[0.1, 1.0]. Thus, we obtained the layer $L_{ex-cand}$ (see Figure 5d). Finally, to obtain the binary mask of potential EX candidates, $M_{ex-cand}$, we binarized $L_{ex-cand}$. The obtained layers of interest in this phase, $L_{chor-bri}$ (Figure 5b), $L_{bm}$ (Figure 5c), and $L_{ex-cand}$ (Figure 5d), were also useful for lesion candidate classification in later stages. 

### 3.7. Red Lesion Classification

Once the RL candidates were obtained, we used an MLP to separate the true RLs from non-RL candidates. This type of neural network has been used in previous studies for the automatic detection of RLs [17,48]. This stage comprises three steps:

#### 3.7.1. Feature Extraction

For each region candidate in $M_{lr-cand}$, a set of features was extracted using the previously obtained layers. We included 100 features, as specified in Table 1. As it can be seen, most of the features are directly extracted from the decomposed layers. 

#### 3.7.2. Feature Selection

Reducing the number of features to a set of relevant, low-correlated ones decreases classification errors and simplifies the structure of the classifier [40]. For this task, the FCBF method was applied [39]. FCBF is classifier-independent and computes symmetrical uncertainty to find the most relevant and non-redundant features for a certain problem [39]. The 24 selected features are also specified in Table 1. Features of different nature were selected, including shape, distance, intensity, and variability around the candidates in different layers.

#### 3.7.3. Multilayer Perceptron Neural Network

An MLP consists of various fully connected layers of neurons. It maps a set of input variables onto a set of output variables using a nonlinear function [40]. Since a single hidden layer of neurons is capable of universal approximation, a 3-layer MLP was used (input-hidden-output) [49]. The number of neurons in the input layer was the number of selected features. We used a single neuron in the output layer, since our problem was dichotomous. The number of hidden units was experimentally optimized during the training stage. The activation function used in the hidden layer was the hyperbolic tangent sigmoid (tanh), which accelerates the learning process of the network [40]. The logistic sigmoid was used as the activation function in the output neuron. We used the scaled conjugate gradient backpropagation method as the learning function. The cross-entropy error function was the selected error function to minimize during training [17,40]. In addition, we used the regularization parameter, experimentally optimized during training, to avoid overfitting and improve generalization [40].

### 3.8. Exudate Classification

After EX candidate segmentation, we used the MLP to detect the true EX. This type of network has also been used in previous studies for the automatic detection of EXs [50]. This classification stage comprises three steps:

#### 3.8.1. Feature Extraction

For each region candidate in $M_{ex-cand}$, a set of features was extracted using the layers obtained in the previous stages. It should be noted that the same set of 100 features used for RL candidate classification were also used in the case of EXs. They are collected in Table 1.

#### 3.8.2. Feature Selection

As for the RL classification, we used the FCBF method to select a reduced number of features. The 34 selected features in this stage are specified in Table 1. Again, features of a different nature were selected, including shape, distance, intensity, and variability around the candidate in different layers.

#### 3.8.3. Multilayer Perceptron Neural Network

For this step, EX candidates were classified using an MLP with the same configuration as the one used for RL candidate classification. However, a joint classification for both types of lesions would not be possible in our approach, since the selected features were different. Moreover, it is interesting to separately classify the lesions from a clinical point of view. They often appear at different times and have implications in determining the severity of the disease. In this case, the number of neurons in the input layer was the number of selected features in the previous step. The number of neurons in the hidden layer and regularization parameter were also experimentally optimized during the training process.

### 3.9. Performance Assessment for Lesion Detection

All optimal values for the parameters of the proposed method were obtained using the 281 images from the training set of the proprietary database. We obtained the final results using the test set of the proprietary database (283 images) and the test set of the DiaretDB1 database (61 images). For this purpose, two criteria were considered:

#### 3.9.1. Pixel-Based Criterion

A lesion was correctly identified when, at least one of its pixels was detected [17]. In this case, the lesion was considered as correctly detected. For this criterion, we calculated the pixel-based positive predictive value ($PPV_{p}$) and sensitivity ($SE_{p}$).

#### 3.9.2. Image-Based Criterion

An image was considered pathological when a minimum number of pixels were detected as lesions [17]. In this work, this minimum value was set to 30 pixels [17,51]. Automatic detections of a lower number of pixels were interpreted as noise, since they represent a very small fraction of the image (<0.00001%). Based on the image-based criterion, the average sensitivity ($SE_{i}$), specificity ($SP_{i}$), and accuracy ($ACC_{i}$) were computed.

## 4. Results

### 4.1. Red Lesion Detection

We extracted 4889 RL candidates from the training set. Only 2029 of them were true RL. We randomly selected another 2029 non-RL regions to balance the two classes. The extracted features over the RL candidates were normalized (mean = 0, standard deviation = 1) to improve the classification results [40].

#### 4.1.1. MLP Configuration on the Training Set

**Figure 6:**
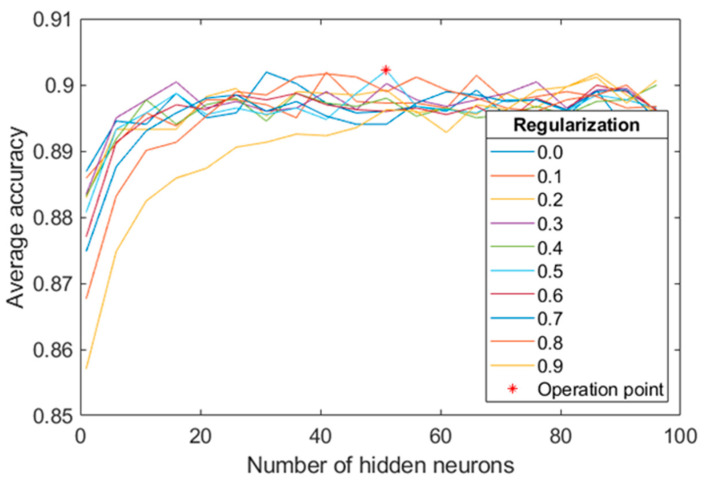
Average accuracy for RL classification over the validation set obtained during MLP training for varying the number of hidden neurons and the regularization parameter.

We experimented with the number of hidden neurons in the range [1:1:100]. The regularization parameter values were varied in the range [0:0.1:1]. For this task, we applied 10-fold-cross-validation exclusively using the training set of the proprietary database. This technique is a powerful preventative measure against overfitting. The chosen values for those parameters were 51 and 0.5, respectively, since they maximized the average accuracy over the validation test (see Figure 6). 

#### 4.1.2. Red Lesion Detection on the Test Set

The results for RL detection in terms of the pixel-based criterion and image-based criterion using both the proprietary database and the public database can be seen in Table 2. These results were obtained by applying the criteria in Section 3.9 and show the performance of the complete algorithm.

### 4.2. Exudate Detection

We extracted 4782 EX candidates from the training set. Only 2072 of them were true EX. Thus, we randomly selected another 2072 non-EX regions to balance the two classes. The extracted features over the EX candidates were also normalized to improve the classification results (mean = 0, standard deviation = 1) [40].

#### 4.2.1. MLP Configuration on the Training Set 

**Figure 7:**
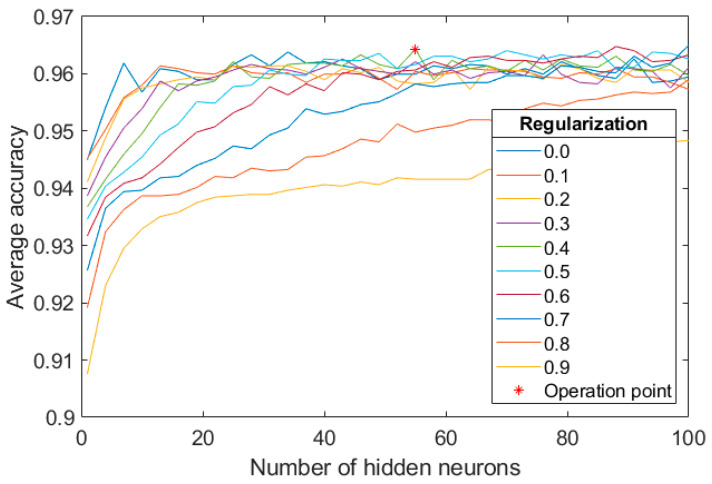
Average accuracy for EX classification over the validation set obtained during MLP training for varying the number of hidden neurons and the regularization parameter.

As for the RL classification, we varied the number of hidden neurons in the range [1:1:100] and the regularization parameter values in the range [0:0.1:1]. We applied 10-fold-cross-validation exclusively using the training set of the proprietary database allowing to control overfitting. The chosen value for those parameters was 55 and 0.4, respectively, since they maximized the average accuracy over the validation test (see Figure 7). 

#### 4.2.2. Exudate Detection on the Test Set

The results for EX detection in terms of pixel-based criterion and image-based criterion using both the proprietary database and the public database are presented in Table 3. These results were obtained by applying the criteria in Section 3.9 and show the performance of the complete algorithm.

## 5. Discussion

In this study, we have proposed automatic methods for the detection of DR-related retinal lesions. The fundus image was decomposed into various layers representing different structures of the retina, which is the main contribution of this paper. Among these layers, the lesion candidates, the choroidal vasculature visible in tigroid retinas, and the reflective features were included, having proved useful for the classification of retinal lesions. To the best of our knowledge, there are no previous studies that have decomposed the image separating the relevant retinal structures such as the choroidal vessels and the reflective features to detect RLs and EXs.

The proposed method for retinal lesion detection was evaluated on a set of 283 fundus images. Among them, 120 showed RLs, 92 showed EXs, and 76 images showed both types of lesions. The proprietary database was very heterogeneous, showing variations in color, luminosity, contrast, and quality among images. In the same way, variable lesions in terms of appearance and size could be found. The method was also evaluated on the test set of the public database DiaretDB1, composed of 61 images. The results for the detection of RLs and EXs were measured using a pixel-based criterion and an image-based criterion. Results can be seen in Table 2 and Table 3. 

All of these results can be compared to those obtained in previous studies according to the image-based criterion, as shown in Table 4 and Table 5. However, comparisons should be made with caution, since the databases and the performance measures vary among studies. We have found four methods that have been evaluated using the DiaretDB1 database in order to establish a direct comparison with the proposed method for RL detection. Jaafar et al. [52] obtained a high $SE_{i}$ = 98.80%, yet a $SP_{i}$ = 86.20% lower than ours ($SP_{i}$ = 91.67%). In addition, they tested their method using the database DiaretDB0 together with DiaretDB1. In the work of Roychowdhury et al. [53], they obtained $SP_{i}$ = 93.73%, but the $SE_{i}$ = 75.50% was low. In [16], $SP_{i}$ = 91.67% was obtained. However, our value of $SE_{i}$ (88.00%) improves their $SE_{i}$ (83.30%). Table 4 also shows that the proposed method achieves better results than our previous work [17]. Regarding EX detection, we have also found several methods that have been assessed using the DiaretDB1 database. Walter et al. [27] obtained $SE_{i}$ = 86.00% and $SP_{i}$ = 69.00%. In [54], values of $SE_{i}$ = 92.00% and $SP_{i}$ = 68.00% were obtained. Liu et al. [55] achieved $SE_{i}$ = 83.00% and $SP_{i}$ = 75.00%. The method proposed in [32] showed a $SE_{i}$ = 88.00% and $SP_{i}$ = 95.00%. Kaur and Mittal obtained $SE_{i}$ = 91.00% and $SP_{i}$ = 94.00%. Finally, the work of Adem et al. presented high values of $SE_{i}$ = 99.20% and $SP_{i}$ = 97.97%. The value of $SE_{i}$ achieved with our method (95.00%) is higher than those obtained in previous studies, with only one exception [33]. It should be noted that the test set in [33] was composed of images from DiaretDB0 and DRIMDB databases in addition to DiaretDB1. Moreover, the training set of the DiaretDB1 database was used in the training phase and the appearance of these images is similar to the ones in the test set of the same database. Therefore, this could make obtaining good results easier. When comparing our results with previous approaches, it should be noted that the proposed method has been developed using only images from the proprietary database. This dataset is different from the public database DiaretDB1 in several aspects. Firstly, the images in our database have a higher resolution. Secondly, they have been captured using a different protocol. Thirdly, the FOV in the images of the proprietary database is 45°, while the FOV in the images of DiaretDB1 is 50°. In addition, they were selected considering different quality criteria [37]. In spite of these differences, the results on the test set of DiaretDB1 prove the robustness of the proposed method. 

Our study also has some limitations that should be mentioned. First, segmenting the blood vessels as an independent stage has some drawbacks. When an RL is detected as part of the vascular network, it is discarded as a possible lesion, regardless of the later stages. To avoid this problem, the elimination of the blood vessel segments could be addressed in the last stage using supervised classification. Second, some parameters of the method were empirically set. However, the image normalization achieved in the preprocessing stage makes the value of those parameters effective for any input image. Furthermore, the value of these parameters is not critical, and the performance is not significantly affected as long as they are around the selected values. We noticed that small deviations of these values hardly produced changes in the output. This is especially true when it comes to the saturation channel (S) of HSV. Thus, we could declare that the values for the channels hue (H) and value (V) could be deviated around the range [−0.03, 0.03] producing a similar segmentation, while the saturation channel could be deviated around the range [−0.05, 0.05] to obtain almost the same result. Third, the classification stage is based on a set of handcrafted features. Even though they have proved to represent lesions adequately with a moderate database size, we intend to explore deep-learning-based approaches in future studies. These methods will require that we also increase the size of the database for proper training.

## 6. Conclusions

In this work, we have proposed a method for the detection of RLs and EXs in fundus images. The method has been developed based on the clues observed by the ophthalmologists to identify the different structures of the retina. This allowed us to study the existing relation between the lesions and other structures, such as the choroidal vessels and the reflective features. The extracted layers using our decomposition method have proven to be useful to detect DR-related retinal lesions. Our results suggest that the proposed method could be used as part of an automatic DR screening system. Thus, it could be a diagnostic aid for the early detection of DR, reducing the workload of specialists and improving the management of diabetic patients.

## Figures and Tables

**Table 1:** Extracted features for lesion classification.

Num.	Description	Selected for Red Lesion (RL) Detection	Selected for Exudate (EX) Detection
1	Area of the region	-	-
2	Width of the bounding box (smallest rectangle containing the region)	-	-
3	Height of the bounding box	-	-
4	Area of the smallest convex hull (smallest convex polygon that can contain the region)	-	-
5	Eccentricity of the ellipse that has the same second moments as the region	5	5
6	Number of holes in the region	-	-
7	Ratio of pixels in the region to pixels in the total bounding box	-	7
8	Length of the major axis of the ellipse that has the same normalized second central moments as the region	-	-
9	Length of the minor axis of the ellipse that hast the same normalized second central moments as the region	-	-
10	Distance around the boundary of the region (perimeter length)	-	-
11	Proportion of the pixels in the convex hull that are also in the region (solidity)	11	-
12–14	Mean of the pixels inside the region computed in the Red-Green- Blue (RGB) channels of the image $I_{prep}$	13	-
15–17	Median of the pixels inside the region computed in the RGB channels of the image $I_{prep}$	-	17
18–20	Standard deviation of the pixels inside the region computed in the RGB channels of the image $I_{prep}$	18, 19	18–20
21–23	Entropy of the pixels inside the region computed in the RGB channels of the image $I_{prep}$	22, 23	21–23
24–26	Mean of the pixels inside the region computed in the Hue-Saturation-Value (HSV) channels of the image $L_{rl-cand}$/$L_{ex-cand}$	24, 26	26
27–29	Median of the pixels inside the region computed in the HSV channels of the image $L_{rl-cand}$/$L_{ex-cand}$	28, 29	27, 29
30–32	Standard deviation of the pixels inside the region computed in the HSV channels of the image $L_{rl-cand}$/$L_{ex-cand}$	32	30, 32
33–35	Entropy of the pixels inside the region computed in the HSV channels of the image $L_{rl-cand}$/$L_{ex-cand}$	35	34, 35
36–38	Mean of the pixels inside a circle with radius $R_{DO}$ centered on the region computed in the HSV channels of the image $L_{rl-cand}$	-	-
39–41	Median of the pixels inside a circle with radius $R_{DO}$ centered on the region computed in the HSV channels of the image $L_{rl-cand}$	-	-
42–44	Standard deviation of the pixels inside a circle with radius $R_{DO}$ centered on the region computed in the HSV channels of the image $L_{rl-cand}$	44	42
45–47	Entropy of the pixels inside a circle with radius $R_{DO}$ centered on the region computed in the HSV channels of the image $L_{rl-cand}$	-	-
48–50	Mean of the pixels inside a circle with radius $R_{DO}$ centered on the region computed in the HSV channels of the image $L_{chor-dark}$	-	-
51–53	Median of the pixels inside a circle with radius $R_{DO}$ centered on the region computed in the HSV channels of the image $L_{chor-dark}$	-	-
54–56	Standard deviation of the pixels inside a circle with radius $R_{DO}$ centered on the region computed in the HSV channels of the image $L_{chor-dark}$	-	-
57–59	Entropy of the pixels inside a circle with radius $R_{DO}$ centered on the region computed in the HSV channels of the image $L_{chor-dark}$	59	57
60–62	Mean of the pixels inside a circle with radius $R_{DO}$ centered on the region computed in the HSV channels of the image $L_{chor-bri}$	-	62
63–65	Median of the pixels inside a circle with radius $R_{DO}$ centered on the region computed in the HSV channels of the image $L_{chor-bri}$	63–65	64, 65
66–68	Standard deviation of the pixels inside a circle with radius $R_{DO}$ centered on the region computed in the HSV channels of the image $L_{chor-bri}$	66	-
69–71	Entropy of the pixels inside a circle with radius $R_{DO}$ centered on the region computed in the HSV channels of the image $L_{chor-bri}$	-	-
72–74	Mean of the pixels inside a circle with radius $R_{DO}$ centered on the region computed in the HSV channels of the image $L_{ex-cand}$	-	73, 74
75–77	Median of the pixels inside a circle with radius $R_{DO}$ centered on the region computed in the HSV channels of the image $L_{ex-cand}$	-	-
78–80	Standard deviation of the pixels inside a circle with radius $R_{DO}$ centered on the region computed in the HSV channels of the image $L_{ex-cand}$	-	78–80
81–83	Entropy of the pixels inside a circle with radius $R_{DO}$ centered on the region computed in the HSV channels of the image $L_{ex-cand}$	-	83
84–86	Mean of the pixels inside a circle with radius $R_{DO}$ centered on the region computed in the HSV channels of the image $L_{bm}$	-	-
87–89	Median of the pixels inside a circle with radius $R_{DO}$ centered on the region computed in the HSV channels of the image $L_{bm}$	-	-
90–81	Standard deviation of the pixels inside a circle with radius $R_{DO}$ centered on the region computed in the HSV channels of the image $L_{bm}$	90	91
93–95	Entropy of the pixels inside a circle with radius $R_{DO}$ centered on the region computed in the HSV channels of the image $L_{bm}$	-	93
96	Mean of all the pixels the V channel of the image $L_{bm}$	96	96
97	Mean of the pixels calculated in the border of the region applying Prewitt operator in the image $I_{prep}$	97	97
98	Mean of the pixels inside the region calculated in the result of applying multiscale line operator filters	98	98
99	Distance to the center of the optic disc (OD)	-	99
100	Distance to the center of the fovea	100	100

**Table 2:** Results for the detection of red lesions.

Database	Pixel-Based Criterion		Image-Based Criterion		
	$SE_{p}$	$PVV_{p}$	$SE_{i}$	$SP_{i}$	$ACC_{i}$
Proprietary	82.25	91.07	85.00	90.80	88.34
DiaretDB1	84.79	96.25	88.00	91.67	90.16

**Table 3:** Results for the detection of exudates.

Database	Pixel-Based Criterion		Image-Based Criterion		
	$SE_{p}$	$PPV_{p}$	$SE_{i}$	$SP_{i}$	$ACC_{i}$
Proprietary	89.42	96.01	88.04	98.95	95.41
DiaretDB1	91.65	98.59	95.00	90.24	91.80

**Table 4:** Comparison of some methods for red lesion detection.

Method	Database	Nb.	$SE_{i}$	$SP_{i}$
Jaafar et al., 2011 [52]	DiaretDB1	219	98.80	86.20
Roychowdhury et al., 2012 [53]	DiaretDB1	89	75.50	93.73
Zhou et al., 2017a [16]	DiaretDB1	89	83.30	97.30
Romero-Oraá et al., 2019 [17]	DiaretDB1	89	84.00	88.89
García et al., 2010 [51]	Private	115	100	56.00
Niemeijer et al., 2005 [56]	Private	100	100	87.00
Grisan and Ruggeri, 2005 [57]	Private	260	71.00	99.00
Seoud et al., 2016 [15]	Messidor	1200	83.30	97.30
Orlando et al., 2018 [19]	Messidor	1200	91.10	50.00
Sánchez et al., 2011 [58]	Messidor	1200	92.20	50.00
**Proposed method**	**DiaretDB1**	**89**	**88.00**	**91.67**

**Table 5:** Comparison of some methods for exudate detection.

Method	Database	Nb.	$SE_{i}$	$SP_{i}$
Walter et al., 2002 [27]	DiaretDB1	89	86.00	69.00
Harangi and Hajdu, 2014 [54]	DiaretDB1	89	92.00	68.00
Liu et al., 2016 [55]	DiaretDB1	89	83.00	75.00
Zhou et al., 2017b [32]	DiaretDB1	89	88.00	95.00
Kaur and Mittal, 2018 [59]	DiaretDB1	89	91.00	94.00
Adem, 2018 [33]	DiaretDB1	89	99.20	97.97
**Proposed method**	**DiaretDB1**	**89**	**95.00**	**90.24**
